# Stepwise photosensitized thymine dimerization mediated by an exciton intermediate

**DOI:** 10.1007/s00706-017-2108-4

**Published:** 2017-12-04

**Authors:** Clemens Rauer, Juan J. Nogueira, Philipp Marquetand, Leticia González

**Affiliations:** Institute of Theoretical Chemistry, Faculty of Chemistry, University of Vienna, Vienna, Austria

**Keywords:** DNA, Thymine dimerization, Quantum chemical calculations, Non-adiabatic dynamics, Wavefunction analysis, Charge transfer

## Abstract

**Abstract:**

Cyclobutane thymine dimerization is the most prominent DNA photoinduced damage. While the ultrafast mechanism that proceeds in the singlet manifold is nowadays well established, the triplet-state pathway is not completely understood. Here we report the underlying mechanism of the photosensitized dimerization process in the triplet state. Quantum chemical calculations, combined with wavefunction analysis, and nonadiabatic molecular dynamics simulations demonstrate that this is a stepwise reaction, traversing a long-lived triplet biradical intermediate, which is characterized as a Frenkel exciton with very small charge-transfer character. The low yield of the reaction is regulated by two factors: (i) a relatively large energy barrier that needs to be overcome to form the exciton intermediate, and (ii) a bifurcation of the ground-state potential-energy surface that mostly leads back to the Franck–Condon region because dimerization requires a very restricted combination of coordinates and velocities at the event of non-radiative decay to the ground state.

**Graphical abstract:**

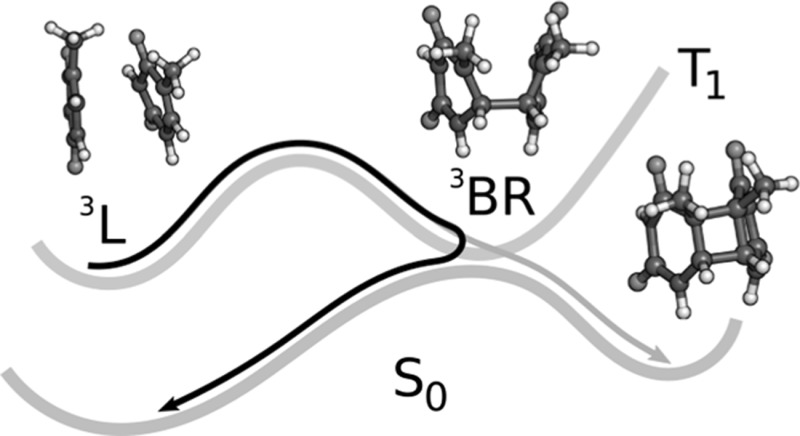

## Introduction

The formation of cyclobutane thymine T〈〉T dimers between two adjacent thymine bases is the most frequent DNA damage under UV radiation [1]. This photolesion, which can take place in both the singlet and triplet manifolds, has been extensively investigated spectroscopically [2–7] and computationally [8–15]. The triplet pathway is a much slower process [7] and exhibits a smaller yield [6, 16] than the singlet mechanism due to inefficient intersystem crossing. As a consequence, this pathway yields very weak spectroscopic signals that preclude unambiguous statements regarding the mechanism [5–7]. In order to enhance the triplet signals, photosensitization is commonly used, increasing the T〈〉T dimerization yield [5, 17–19]. This enhancement can also play a role with photosensitizers acting as phototoxic drugs [20]. Photosensitization involves intersystem crossing of a photosensitizer after excitation, transferring its electronic energy to a neighboring thymine, which is then promoted to the lowest triplet state.

**Fig. 1:**
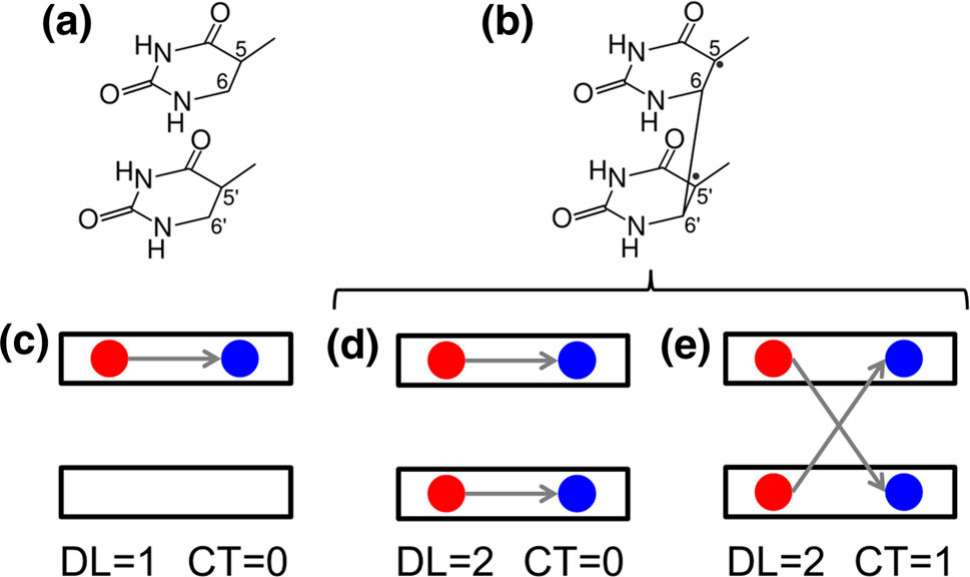
Chemical formula and electronic arrangement of two thymines for **a** the local triplet state (^3^L) and **b** biradical triplet states (^3^BR). Schematic representation of **c** a local state, **d** a Frenkel exciton state, and **e** a charge-resonance state. The black rectangles represent the thymine monomers. The black arrow connects the hole (red circle) and the electron (blue circle) generated after excitation. Delocalization length (*DL*) and charge-transfer (*CT*) contribution are also indicated (color figure online)

Using the photosensitizer 2′-methoxyacetophenone and the dinucleotide TpT, stationary and time-resolved experiments provided two time constants, 22.5 and 62 ns, for the decay of the TpT in the triplet manifold [5]. These constants have been related to a local triplet state (^3^L, see Fig. 1a, c), which is populated after triplet–triplet energy transfer (TTET) from the photosensitizer, and a biradical triplet state (^3^BR, see Fig. 1b, d, e), which can be formed from ^3^L. Quantum chemical calculations [14] suggested that the T〈〉T dimerization is triggered by the formation of the biradical intermediate, but the barrierless pathway calculated for the transition from ^3^L to ^3^BR is in conflict with the experimental lifetime of 22.5 ns assigned to the ^3^L species. This conflict is likely caused by the use in the theoretical study of a perfectly stacked geometrical configuration with *C*_s_ symmetry, which is hardly achieved in a DNA strand or in a TpT dimer due to the geometrical constraints of the sugar-phosphate backbone. Recent quantum mechanics/molecular mechanics (QM/MM) calculations have found a small barrier of 0.15 eV separating the ^3^L and ^3^BR minima, in better agreement with the experimental lifetime of 22.5 ns assigned to the ^3^L species [15].

An intriguing question in the dimerization process is the character of the ^3^BR state. Calculations showed that the excited electronic density of ^3^BR is distributed over the two thymine units [14] and spectroscopic measurements suggested that dimerization involves the participation of delocalized triplet states [18]. However, electronic delocalization over the two monomers can correspond to two different electronic states: (i) a Frenkel exciton, in which two local excitations are coupled (Fig. 1d), or a charge-resonance state, in which two charge-transfer states with charge flow in opposite directions are combined (Fig. 1e) [21]. It has been speculated that the triplet state involved in dimerization could be a charge-transfer state [19], as theoretically predicted for the thymine–thymine 6-4 adduct formation [22]. However, evidence of charge-transfer states for the T〈〉T dimerization has never been reported. An additional unsolved mechanistic feature is the reason behind the very low yield of dimerization even when the triplet manifold is forced to be populated after triplet–triplet energy transfer from a photosensitizer.

In this paper, we use quantum chemical calculations, wavefunction analysis, and nonadiabatic surface-hopping molecular dynamics simulations to provide a clear-cut mechanism for the photosensitized thymine dimerization. We study the formation of the ^3^BR precursor electronic triplet state from the ^3^L state and identify the nature of these species in terms of electronic delocalization and charge-transfer character. Furthermore, we offer a rationale for the factors behind the small quantum yield of the reaction.

## Results and discussion

**Fig. 2:**
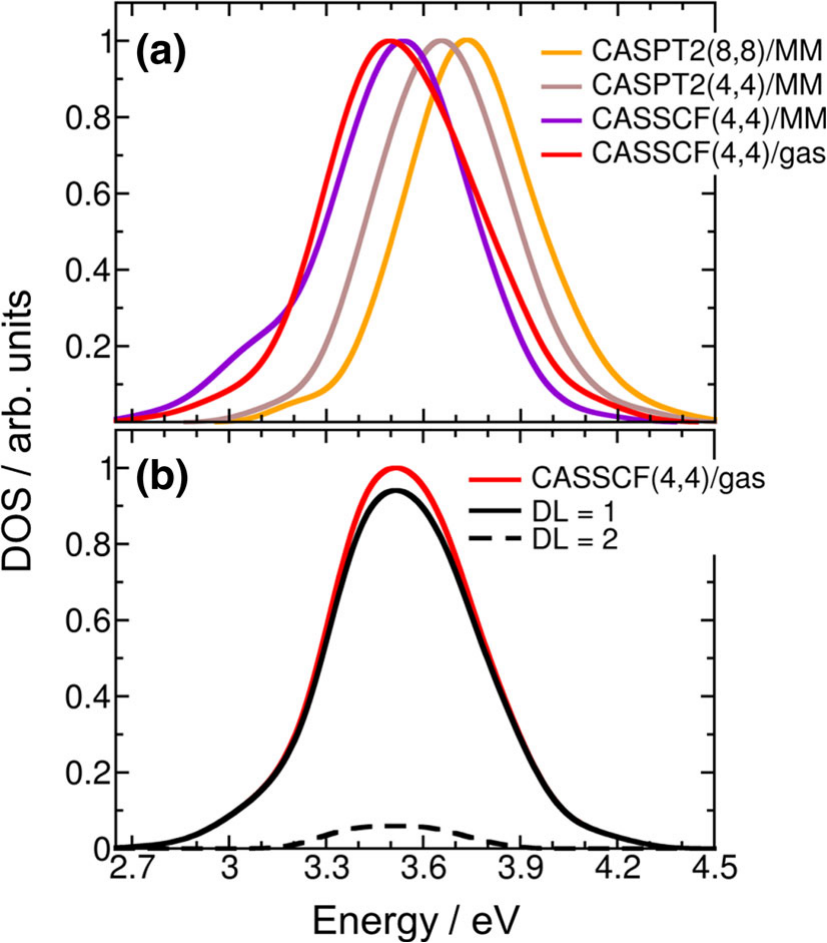
**a** Lowest-energy band of density of triplet states for the thymine–thymine stacked pair embedded in a solvated single strand (dT)_12_ computed at MS(3)-CASPT2(8,8)/MM, MS(3)-CASPT2(4,4)/MM, SA(3)-CASSCF(4,4)/MM, and SA(3)-CASSCF(4,4)/gas phase levels of theory and for the thymine–thymine stacked pair in the gas phase computed at SA-CASSCF level of theory. **b** Delocalization length (*DL*) decomposition of the SA(3)-CASSCF(4,4) density of triplet states of the thymine–thymine stacked pair in the gas phase

The first step of our study is to select the level of theory for the electronic-structure calculations, especially for the nonadiabatic surface-hopping dynamics simulations. We commence by computing the lowest-energy band of the density of triplet states, which involves the *T*_1_ and *T*_2_ electronic states, of a thymine–thymine stacked pair embedded in a solvated single strand (dT)_12_. Triplet excitation energies were calculated with an electrostatic QM/MM [23] scheme where the two nucleobases in the middle of the strand were described by multistate complete active space second-order perturbation [24] (MS-CASPT2) theory and the rest of the system by a force field [25, 26]. The QM region was also described by state-average complete active space self-consistent field (SA-CASSCF) [27] to investigate whether dynamical correlation is necessary to describe the lowest-energy triplet band. In addition, the MS-CASPT2/MM calculations have been performed employing two different active spaces, namely (4,4) and (8,8). The first one only includes the four *π* orbitals and the four electrons involved in the dimerization reaction (orbitals *π*_3_, *π*_4_, *π*_5_*, and *π*_6_* in Fig. 9). The second active space has two additional electrons and two additional *π* orbitals for each nucleobase. The calculations were performed on an ensemble of 250 geometries taken from a previous ground-state QM/MM molecular dynamics simulation [12]. The density-of-states bands computed at the different levels of theory are plotted in Fig. 2a. The MS-CASPT2(8,8)/MM band is blue shifted by only 0.08 eV with respect to the MS-CASPT2(4,4)/MM one. This means that the smaller active space is enough to describe most of the static correlation. The small energy difference of 0.12 eV between MS-CASPT2(4,4)/MM and SA-CASSCF(4,4)/MM shows that a correct qualitative picture can be obtained without including dynamical correlation in the calculation. The electrostatic effect of the solvated DNA environment in the triplet excited states is small. This can be seen by comparing the SA-CASSCF(4,4)/MM and SA-CASSCF(4,4)/gas phase bands, whose energy maxima differ only  by 0.04 eV. Overall, the energy difference between the highest level of theory [MS-CASPT2(8,8)/MM] and the lowest level of theory [SA-CASSCF(4,4) in the gas phase] employed here is 0.24 eV. Therefore, based on these results at the Franck–Condon region, SA-CASSCF calculations in the gas phase seem to be suitable to describe the lowest-energy triplet states of the thymine dimer embedded in a DNA strand.

The first step of the reaction is the population of T_1_ after TTET. The character of the T_1_ state can either be ^3^L or ^3^BR depending on its electronic configuration (see Fig. 1). For most of the geometries within the Franck–Condon region, it is expected that the *T*_1_ electronic state corresponds to the locally excited configuration ^3^L as the relatively large rise distance (3.5 Å) between stacked nucleobases in DNA strands mostly precludes the direct formation of ^3^BR state, where the C_6_–C_6_′ bond is already preformed. The excited electronic density in ^3^L is completely located at one of the thymine nucleobases (Fig. 1c), while ^3^BR has the spin density equally distributed over the ethylenic C_5_ and C_5_′ atoms of both thymine bases (Fig. 1d, e). Since the C_6_–C_6_′ bond is already preformed in the ^3^BR species, it is likely that the T〈〉T dimerization is triggered by the formation of the biradical intermediate, as suggested in the literature [5, 14].

Even if the C_6_–C_6_′ bond is not preformed within the Franck–Condon region, we found it interesting to investigate whether any initial geometrical configuration presents ^3^BR character. To this aim, we analyzed the electronic transition density [21, 28, 29] of the triplet states that compose the density of states, from which the delocalization length (*DL*), defined as the number of nucleobases involved in the excitation process [30], was computed. For ^3^L, the excitation is localized in only one of the thymine bases (*DL* = 1), while in ^3^BR both thymine monomers are involved in the excitation (*DL* = 2). Figure 2b shows the calculated density of triplet states in the gas phase decomposed by delocalization length. We find that the lowest-energy triplet band is mainly composed by local excitations ^3^L, while the contribution of excitations delocalized over the two monomers is very small. Since the photosensitizer employed in the experiments [5] was initially excited at ∼ 4 eV, the calculated states composing this band (between 2.7 and 4.4 eV) are the only ones energetically accessible by triplet–triplet energy transfer. Unequivocally, most of the states populated at the Franck–Condon region are locally excited states, i.e. correspond to the ^3^L triplet state.

**Fig. 3:**
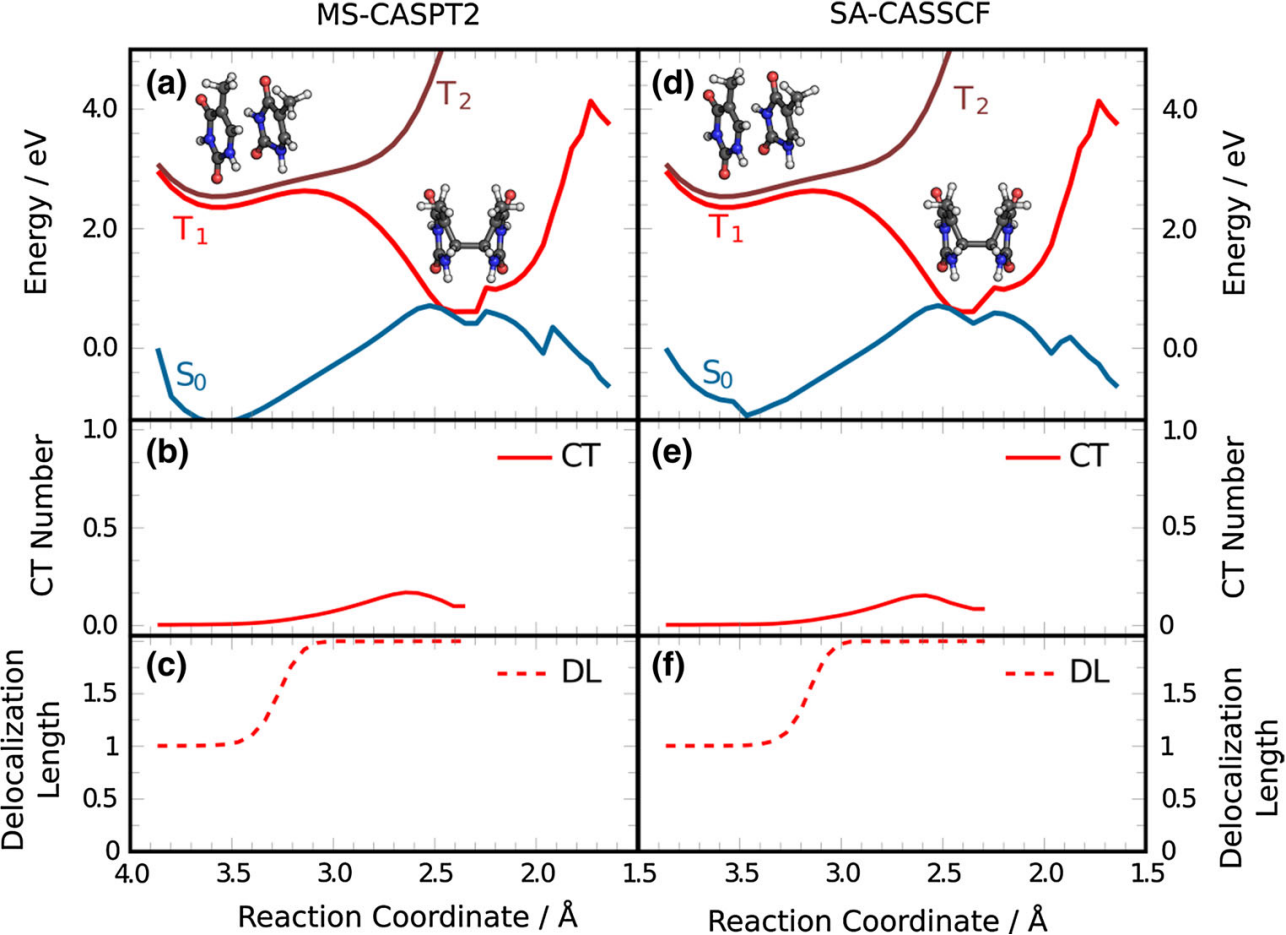
Variation of **a**, **d** the potential energy of *S*_0_, *T*_1_, and *T*_2_, and **b**, **e** charge-transfer (*CT*) contribution and **c**, **f** the delocalization length (*DL*) of *T*_1_ along a linearly interpolated pathway along the reaction coordinate (average of the C_6_–C_6_′ and C_5_–C_5_′ bond lengths) connecting the initial ^3^L structure with the ^3^BR minimum and continuing from there to the thymine dimer. The calculations were carried out for the gas phase employing MS-CASPT2(4,4) and SA-CASSCF(4,4) levels of theory

After having established that the ^3^L state is initially populated, in agreement with spectroscopic measurements, the next step is the formation of the ^3^BR state. Figure 3a shows the MS-CASPT2 energies of the *S*_0_, *T*_1_, and *T*_2_ state in a static scan from the ^3^L state to the ^3^BR minimum and from the ^3^BR minimum to the dimer. In qualitative agreement with the barrier obtained in Ref. [15], a barrier of 0.27 eV separates the ^3^L and the ^3^BR minima in *T*_1_. This energy barrier agrees very well with the barrier of 0.30 eV that is obtained by using the Arrhenius equation at a temperature of 300 K and using the experimental deactivation time of 22 ns [5], despite the approximations taken. The relatively large energy barrier is likely the first reason that explains the low yield of the reaction as in many cases the system has enough time to return to the ground state by intersystem crossing before overcoming the barrier.

The electronic wavefunction of *T*_1_ along the pathway between ^3^L and ^3^BR is analyzed in Fig. 3b, c. Specifically, the delocalization length (*DL*) and the charge-transfer fraction were computed from the electronic transition density [21, 28, 29]. The delocalization length clearly shows that the dimer is in a locally excited state (*DL* = 1) before the barrier and, after overcoming the barrier, it evolves towards the ^3^BR excited state (*DL* = 2). This ^3^BR excited state can be a Frenkel exciton state or a charge-resonance state (recall Fig. 1d, e). Due to the small separation between both thymine monomers at the ^3^BR minimum, the formation of charge-transfer states, favoured by orbital-overlap interactions [31], is possible. Therefore, the ^3^BR state could acquire charge-resonance character along the dimerization pathway. The solid line in Fig. 3b unambiguously shows that the charge-transfer contribution is very small along the path that connects ^3^L with ^3^BR. This demonstrates that ^3^BR is mainly a Frenkel exciton state. Only in the region near to the ^3^BR/*S*_0_ crossing the charge-transfer contribution is around 0.15, indicating that the Frenkel state acquires a small degree of charge-transfer character. This conclusion is in contrast to the hypothesis put forward in Ref. [19], claiming that charge-transfer triplet states could be present in the T〈〉T dimerization. Our calculations clearly demonstrate that the precursor electronic state leading to dimerization is a Frenkel exciton state and not a charge-transfer state. Recent theoretical calculations predicted that T〈〉T dimerization in the singlet manifold is also mediated by an exciton intermediate [8]. Figure 3d–f shows the same energy scan and wavefunction analysis computed at SA-CASSCF level. Since the energy and character of the states are very similar to the ones obtained by MS-CASPT2, as was also the case for the density of states computed at the Franck–Condon region, the subsequent gas-phase dynamics simulations are performed using SA-CASSCF for the electronic-structure calculations.

After the formation of the ^3^BR species, the system is trapped in the ^3^BR minimum (recall Fig. 3a). This minimum coincides with the crossing point with the ground state *S*_0_. Dimerization takes place only when the appropriate region of the *S*_0_ potential is populated after intersystem crossing from *T*_1_. As the experimental [5] decay time constant is 62 ns for ^3^BR, the radiationless decay to the ground state is a very slow process. Once in the ground state, the system can dimerize or return to the reactant region without causing damage. The experimentally determined dimerization yield is only 4% [5]. In order to determine the factors that govern this low yield, we have sampled the ^3^BR minimum of *T*_1_ for at least 100 fs with non-adiabatic surface hopping molecular dynamics simulations in the gas phase using the SHARC code [32].

**Fig. 4:**
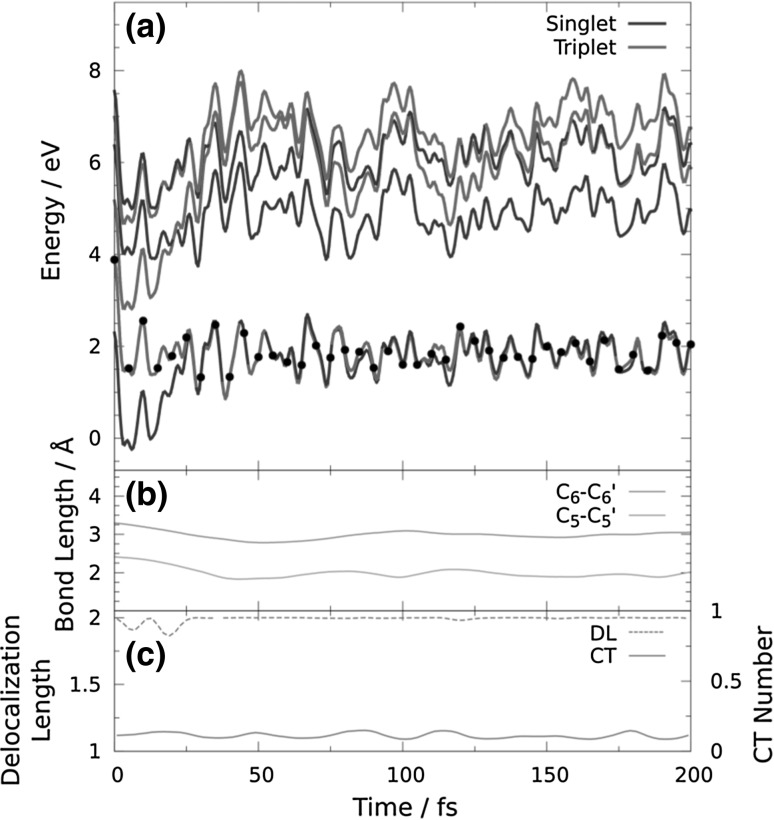
Time evolution of **a** energy levels, **b** the C_5_–C_5_′ and C_6_–C_6_′ distances, and **c** the delocalization length and charge-transfer (*CT*) contribution for a trajectory trapped in the minimum of *T*_1_

**Fig. 5:**
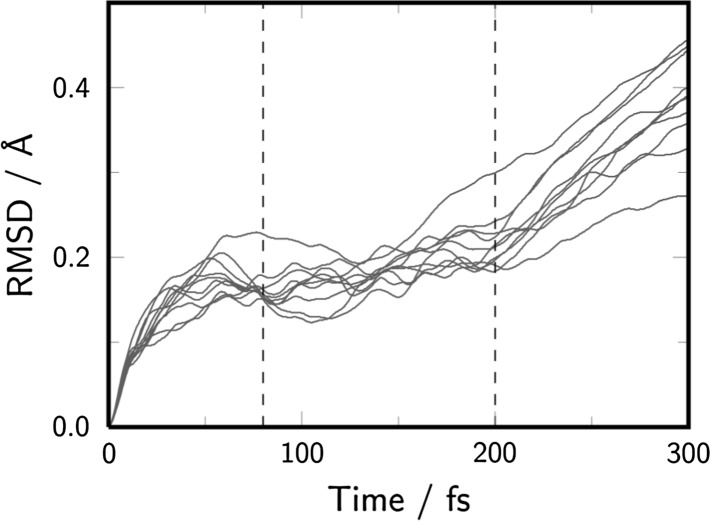
Root-mean squared displacement (RMSD) of the nonadiabatic dynamics trajectories running in the *T*_1_ state. Dashed lines indicate the area from which the geometries were randomly chosen to be manually placed to the ground state

**Fig. 6:**
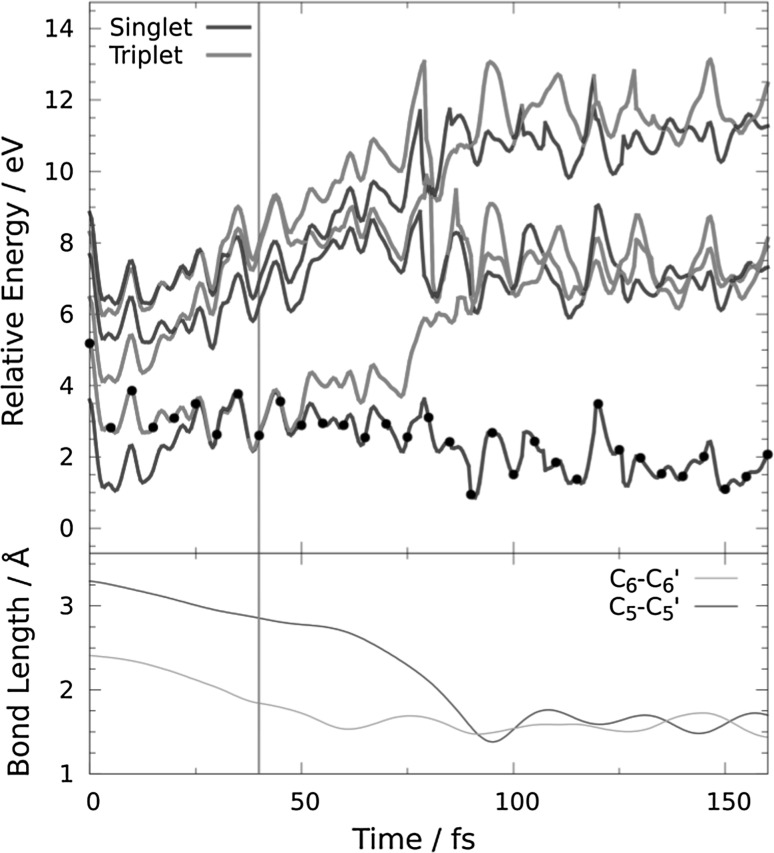
Time evolution of **a** energy levels and **b** the C_5_–C_5_′ and C_6_–C_6_′ distances for a reactive trajectory manually placed in the ground state close to the ^3^BR/*S*_0_ crossing point. The vertical grey line indicates the moment at which the trajectory is transferred to the *S*_0_

As expected, none of the trajectories that sampled the *T*_1_ minimum showed intersystem crossing to the ground state during 100 fs. This is because the spin–orbit coupling around the ^3^BR/*S*_0_ crossing, computed for one of the trajectories as the averaged spin–orbit coupling of 100 snapshots, is merely 1 cm^−1^. Since the intersystem crossing rate depends on the spin–orbit coupling [33], the system can survive in the *T*_1_ minimum for a long time (see Fig. 4 for an example trajectory), in agreement with the large experimental deactivation time of 62 ns [5] and previous calculations [14]. In order to simulate the last step of T〈〉T dimerization, 32 snapshots from the trajectories trapped in the *T*_1_ state were chosen based on a combination of random selection and an ^3^BR/*S*_0_ energy gap smaller than 0.15 eV. At these snapshots, the molecules were manually placed in the ground state, and the dynamics was continued. The selected geometries show an average ^3^BR/*S*_0_ energy gap of 0.07 eV and were taken from the time region of 80–200 fs, based on the root mean squared displacement (RMSD) of the trajectories running in the *T*_1_ state (see Fig. 5). The RMSD shows that at times shorter than 80 fs the geometry of the dimer is not equilibrated and at times longer than 200 fs the nucleobases go apart due to the lack of the sugar-phosphate backbone. Only 5 of these 32 trajectories lead to dimerization, while 27 trajectories returned to the reactant region. Figure 6 displays an example reactive trajectory, in which, first, the C_6_–C_6_′ bond formation is completed after 60 fs, and then, the C_5_–C_5_′ bond is formed after additional 40 fs. The low number of reactive trajectories qualitatively agrees with the low experimental yield of 4% obtained from spectroscopic measurements [5]. However, due to the small number of trajectories employed here, we cannot make any comments on the statistics of the reaction.

**Fig. 7:**
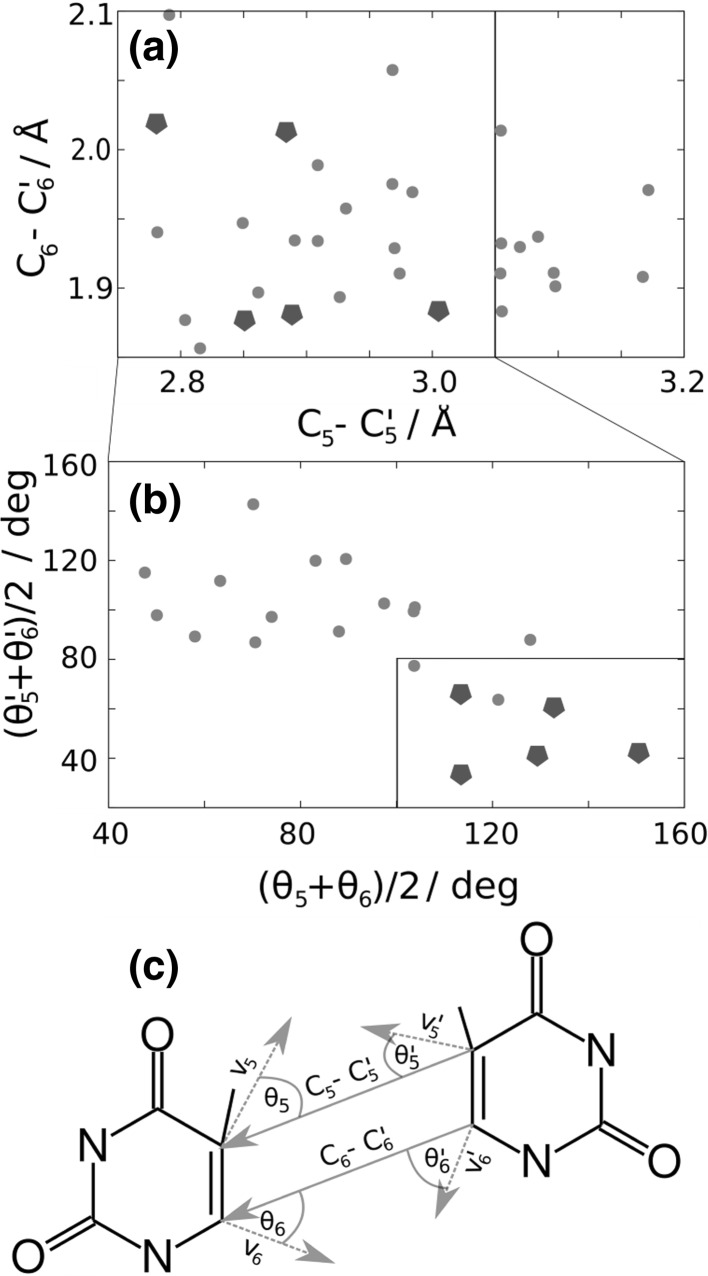
**a** C_5_–C_5_′ and C_6_–C_6_′ distances, **b** average angles formed between the distances C_5_–C_5_′ and C_6_–C_6_′ and the velocities at the atoms C_5_ and C_6_ at the moment of the ^3^BR→*S*_0_ transition for 32 trajectories. **c** Definition of distances and angles employed in the analysis. The lines across **a**–**c** indicate that the next panel is a subset of the data points marked in the box of the previous panel. The green pentagons indicate trajectories undergoing dimerization (color figure online)

The low dimerization yield is rationalized by analyzing the space of coordinates and velocities (phase space) at the moment of the ^3^BR/*S*_0_ transition [34, 35]. Note that these ^3^BR/*S*_0_ transitions are approximated by the selection process described above. The relevant internal coordinates that drive the reaction are the C_5_–C_5_′ and C_6_–C_6_′ distances, and the relevant velocities are those of the atoms involved in these distances. In Fig. 7a, b, the values of the C_6_–C_6_′ and C_5_–C_5_′ distances and average angle formed by the velocity vectors of the atoms C_5_ and C_5_′ with the C_5_–C_5_′ vector (*θ*_5_ and *θ*_5_′), and by the velocity vectors of the atoms C_6_ and C_6_′ with the C_6_–C_6_′ vector (*θ*_6_ and *θ*_6_′), are plotted at the moment of the ^3^BR/*S*_0_ transition. Those trajectories that underwent dimerization are represented by green pentagons. Only when the C_6_–C_6_′ and C_5_–C_5_′ distances are lower than 2.1 and 3.0 Å, respectively, dimerization takes place. In addition, the C atoms of each monomer also need to move towards each other with a large degree of directionality, as indicated by the restricted values of the *θ* angles [larger than 100° for (*θ*_5_ + *θ*_6_)/2 and smaller than 80° for (*θ*_5_′ + *θ*_6_′)/2]. Therefore, although the phase space of the system is very wide, due to the large number of degrees of freedom of the system, only the population of a very small region of the phase space induces dimerization. This is the second reason that is responsible for the very small dimerization yield.

## Conclusion

**Fig. 8:**
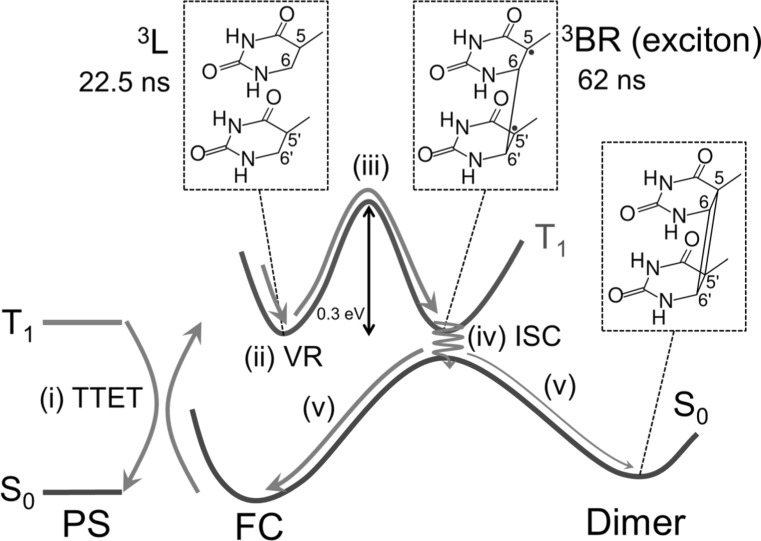
Proposed mechanism of photosensitized T〈〉T dimerization in the triplet state. (i) Triplet–triplet energy transfer (TTET) from the photosensitizer (PS) to thymine–thymine, (ii) vibrational relaxation (VR) in *T*_1_, (iii) ^3^BR formation by overcoming an energy barrier, (iv) intersystem crossing (iv), and (v) formation of thymine dimer or return to the Franck–Condon (FC) region in the electronic ground state

In summary, based on our theoretical results and previous experiments [5], we propose the following stepwise mechanism for the photosensitized T〈〉T dimerization, schematically represented in Fig. 8. First, the locally excited triplet state ^3^L of thymine is populated after triplet–triplet energy transfer from a photosensitizer [step (i) in Fig. 8]. Then, the system vibrationally relaxes to the ^3^L minimum where it stays for 22.5 ns (ii). After overcoming an energy barrier of ca. 0.3 eV (iii), a biradical intermediate ^3^BR with a lifetime of 62 ns is generated within a region that crosses with the electronic ground state. The populated triplet state of the intermediate species is a Frenkel exciton with a small degree of charge-transfer character. Finally, the system undergoes intersystem crossing from *T*_1_ to the ground state (iv), from where it dimerizes with a very small yield, i.e. returning to the initial reactant geometries consisting of two separated thymines in most events (v) due to the tight phase-space restrictions that the system needs to satisfy at the moment of the *T*_1_→*S*_0_ transition.

## Methods

### QM/MM calculation of density of states

The density of states associated to the lowest-energy triplet band of the thymine dimer embedded in a solvated single strand (dT)_12_ and in the gas phase was computed. First, a isothermal-isobaric ensemble (NPT) classical molecular dynamics simulation for solvated (dT)_12_ was evolved at 300 K for 20 ns using the ff14SB [26] and TIP3P [25] force fields to describe DNA and water, respectively. The classical simulation was run with the graphical processing unit (GPU) module pmemd [36] implemented in the Amber14 package [37]. Then, the last snapshot of the classical simulation was taken as the starting one for running quantum mechanics/molecular mechanics (QM/MM) molecular dynamics simulations in the NPT ensemble for 10 ps. The two nucleobases in the middle of the strand were described by the B3LYP functional [38] with D3 dispersion correction [39] and the 6-31G* basis set [40, 41] using the GPU-based code TeraChem1.9 [42, 43] through the interface to external QM programs implemented in Amber14 [37]. More computational details about the molecular dynamics simulations can be found in Ref. [12].

**Fig. 9:**
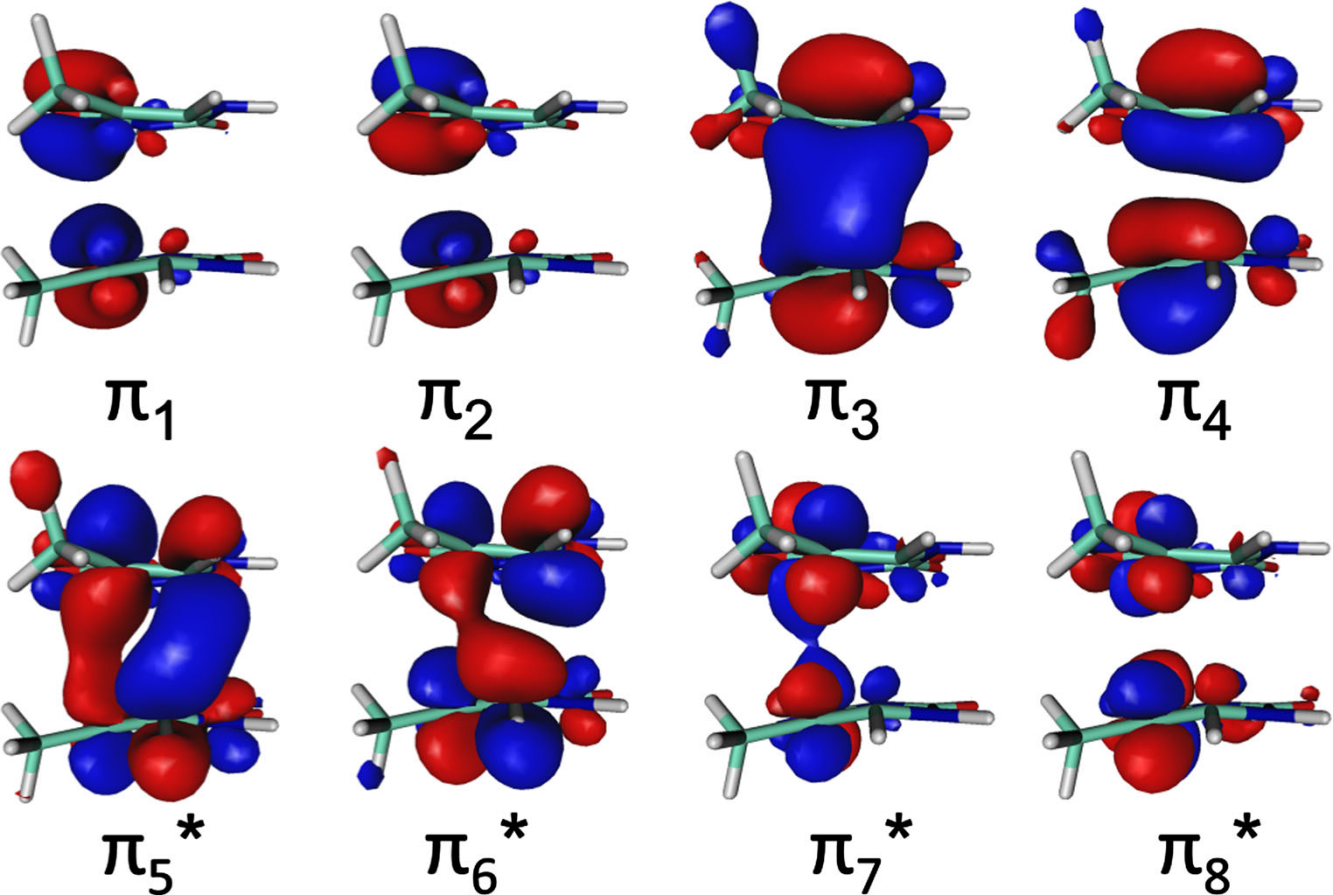
Active orbitals included in the MS-CASPT2/SA-CASSCF(8,8) calculations. When using MS-CASPT2/SA-CASSCF(4,4) only the *π*_3_, *π*_4_, *π*_5_*, and *π*_6_* are used in the active space

An ensemble of 250 equidistant snapshots was selected from the last 5 ps of the QM/MM molecular dynamics simulation. For each snapshot, the electronic excitation energies of the lowest 3 triplet states were computed using an electrostatic embedding QM/MM scheme. The two nucleobases in the middle of the (dT)_12_ strand are described by state-averaged complete active space self-consistent field [27] (SA-CASSCF) using the cc-pVDZ basis set [44, 45], and also by multistate complete active space second-order perturbation (MS-CASPT2) [24] with the same basis set. To minimize the effect of intruder states the level-shift approach was applied with a real-valued shift of 0.3 a.u. The IPEA shift was set to zero, as it is recommended for organic chromophores [46]. The rest of the DNA strand and the water molecules were described by a force field [25, 26]. In addition, the calculations were performed in the gas phase by removing the environment from the 250 snapshots. The two active spaces considered  in the calculations consist of 8 electrons in 8 orbitals and of 4 electrons in 4 orbitals (see Fig. 9). These calculations were carried out with MOLCAS 8 [28, 47]. The resulting excitation energies were convoluted with Gaussian functions with a full width at half maximum of 0.20 eV. The intensity of the bands was scaled to unity. In addition, all electronic triplet states in the gas phase were classified as local states (^3^L) or biradical states (^3^BR) according to the electronic delocalization length, defined as the number of nucleobases involved in the excitation, computed from the electronic transition density [21, 29, 30]. For ^3^L, the excitation is localized in only one of the thymine bases (*DL* = 1), while in ^3^BR both thymine monomers are involved in the excitation (*DL* = 2). The MS-CASPT2/MM, SA-CASSCF/MM, and SA-CASSCF/gas phase density of states and the delocalization-length decomposition of the gas-phase band are plotted in Fig. 2.

### Energy scan in the *T*_1_ potential energy surface

The static calculations for the potential energy scan (Fig. 3), which goes from the Franck–Condon region to dimer formation, were carried out using MS-CASPT2 (Fig. 3a) and SA-CASSCF (Fig. 3d) with the previously described (4,4) active space and the cc-pVDZ basis set. The ^3^L geometry of the Franck–Condon region was taken from the ground state QM/MM molecular dynamics simulation explained above. Specifically, for every of the 30 snapshots whose vertical energy for *T*_1_ is below 3.5 eV, which corresponds to the maxima of lowest-energy band of the density of states, the static scan was performed. Only the scan with the lowest energy barrier in the *T*_1_ state, which tries to mimic a minimum-energy path calculation, is shown in Fig. 3. The geometry at the crossing point between *S*_0_ and *T*_1_ was taken from Ref. [14]. The energies of the two lowest triplet states were computed along a linearly interpolated pathway between both geometries. From the crossing point a second linearly interpolated pathway was connected to the dimer structure, which was taken from our previous work [12]. Moreover, the charge-transfer contribution and delocalization length were also computed along the interpolated pathway using both MS-CASPT2 (Fig. 3b, c) and SA-CASSCF (Fig. 3e, f) [21, 29].

### Non-adiabatic molecular dynamics simulations

Non-adiabatic molecular dynamics simulations were run to sample the *T*_1_/*S*_0_ degeneracy region, in which the *T*_1_ state presents biradical character. Therefore, an arbitrary initial geometry was built with interatomic C_5_–C_5_′ and C_6_–C_6_′ distances of 3.13 and 2.45 Å, respectively. From this geometry 1000 initial conditions (coordinates and velocities) were generated from a zero-Kelvin Wigner distribution [48] based on ground-state frequencies calculated at second-order Møller-Plesset (MP2) perturbation theory [49] using the cc-pVTZ basis set [44] implemented in MOLPRO [50]. From these 1000 initial conditions, 25 were randomly selected to run dynamics on. All trajectories were initially excited to the *T*_1_ state and ran for at least 100 fs or until they left the *T*_1_/*S*_0_ degeneracy region. As the dynamics starts at a close thymine–thymine distance, it was assumed that the reaction is already in progress at the start of the dynamics. Therefore, the initial velocities of all trajectories were modified so that the center of mass of each monomer moves towards each other at a velocity corresponding to the thermal energy (*k*_B_ *T*) at a temperature of 298 K.

From the trajectories running in the degeneracy region, 32 geometries were chosen based on a combination of random selection as well as an ^3^BR/*S*_0_ energy gap smaller than 0.15 eV and continued to run on the ground state potential energy surface. This approach was necessary as none of the trajectories that ran in *T*_1_ hopped to the ground state during their simulation time. The geometries and velocities for the new trajectories running in *S*_0_ were taken from the point where they manually hopped from the parent trajectory, and the electronic coefficients were adapted to put the population on the ground state.

The dynamics simulations were carried out using the ab initio molecular dynamics program SHARC (surface hopping including arbitrary couplings) [32, 51], which uses a modification of the Tully surface hopping method [52] allowing for treating both singlet and triplet states on the same footing. The time step used for the nuclear motion was 0.5 fs, and the time step for the integration of the time-dependent electronic Schrödinger equation was 0.02 fs. All electronic structure properties (energies, gradients, and couplings) were calculated at the SA-CASSCF level of theory using the above described (4,4) active space and the cc-pVDZ basis set. For both the singlet and the triplet state calculations, 3 states were averaged with equal weights each. The non-adiabatic couplings were calculated from the wavefunction overlaps by using a local-diabatization scheme [53]. Additionally, this procedure monitors the wavefunction phase and makes sure that it is maintained throughout the dynamics [54]. Moreover, the Persico decoherence correction [55], with a decoherence parameter of 0.1 a.u. was employed. To save computational time, the gradients of not-populated states were only calculated when their energy was within 0.5 eV of the currently populated state. This procedure is in accordance with previous studies showing that higher lying states only have a minimal effect on the potential of the populated states [32].
